# Microwave Irradiation-Assisted Synthesis of Anisotropic Crown Ether-Grafted Bamboo Pulp Aerogel as a Chelating Agent for Selective Adsorption of Heavy Metals (M^n+^)

**DOI:** 10.3390/gels10120778

**Published:** 2024-11-28

**Authors:** Wenxiang Jing, Min Tang, Xiaoyan Lin, Chai Yang, Dongming Lian, Ying Yu, Dongyang Liu

**Affiliations:** 1 School of Materials and Chemistry, Southwest University of Science and Technology, Mianyang 621010, China; 2 Yibin Forestry and Bamboo Industry Research Institute, Yibin 644005, China; 3 Engineering Research Center of Biomass Materials, Ministry of Education, Southwest University of Science and Technology, Mianyang 621010, China

**Keywords:** crown ether, anisotropic, bamboo pulp aerogel, adsorption

## Abstract

Crown ether is widely used in water purification because of its ring structure and good selective adsorption of specific heavy metals. However, its high cost and difficulty in recycling limit the purification of heavy metals in water. The anisotropic [2,4]-dibenzo-18-crown-6-modified bamboo pulp aerogel (DB18C6/PA) is successfully synthesized by microwave irradiation and directional freezing technology. The physical and chemical properties of DB18C6/PA are analyzed by FTIR, XPS, SEM, TEM, TGA, surface area and porosity analyzers. Single or multivariate systems containing Pb^2+^, Cu^2+^ and Cd^2+^ are used as adsorbents. The effects of the DB18C6 addition amount, pH, initial concentration and adsorption temperature on the adsorption of DB18C6/PA are systematically explored. Pseudo-first-order kinetic models, pseudo-second-order kinetic models and the isothermal adsorption models of Langmuir and Freundlich are used to fit the experimental data. The adsorption selectivity is analyzed from the distribution coefficient and the separation factor, and the adsorption mechanism is discussed. The results show that anisotropic DB18C6/PA has the characteristics of 3D directional channels, high porosity (97.67%), large specific surface area (103.7 m^2^/g), good thermal stability and regeneration (the number of cycles is greater than 5). The surface has a variety of functional groups, including a hydroxyl group, aldehyde group, ether bond, etc. In the single and multivariate systems of Pb^2+^, Cu^2+^ and Cd^2+^, the adsorption process of DB18C6/PA conforms to the pseudo-second-order kinetic model, and the results conform to the Freundlich adsorption isothermal model (a few of them conformed to the Langmuir adsorption isothermal model), indicating that chemical adsorption and physical adsorption are involved in the adsorption process, and the adsorption process is a spontaneous endothermic process. In the single solution system, the maximum adsorption capacities of Pb^2+^, Cu^2+^ and Cd^2+^ by DB18C6/PA are 129.15, 29.85 and 27.89 mg/g, respectively. The adsorption selectivity of DB18C6/PA on Pb^2+^, Cu^2+^ and Cd^2+^ is in the order of Pb^2+^ >> Cu^2+^ > Cd^2+^.

## 1. Introduction

Water pollution is a global environmental problem. With the development of industry and the increasing focus on human health, water pollution has become a major concern [1]. Heavy metal pollution in water bodies is one of the most difficult and harmful pollutants to human life [2]. Moreover, heavy metals can show a high toxicity at low concentrations, which accumulates in the organism for a long time and is difficult to metabolize. As a result, a series of disorders and diseases are caused, which greatly harms human health. There are various methods for treating heavy metals in water bodies. According to the processing principle, these methods include the electrochemical method [3,4], chemical precipitation method [5], membrane treatment method [6], photocatalytic method [7,8], adsorption method [9], etc. Meanwhile, the adsorption method is one of the most popular heavy metal removal methods because of its advantages such as simple technology, rich variety, high efficiency, simple operation, good reversibility, low cost and strong regeneration ability [10,11].

Crown Ethers (CEs) are macrocyclic compounds containing multiple oxygen atoms, and they are named after their structure, which is similar to that of a crown [12,13]. The molecular structure of a CE contains holes, which can complexate with metal cation to form stable complexes. Negatively charged oxygen atoms attract metal cation, and the strength of the host–guest interaction is mainly determined by the size and shape of the crown and the radius of the metal cation. Different CEs have different sizes and shapes of crown ether rings, so they have a significant recognition ability for specific metal ions [14,15]. Crown ether, as a chelating agent of metal cations, has high selective adsorbability during heavy metal adsorption. However, its high cost and difficult recovery have been important factors limiting its use in large-scale process applications [16,17]. Therefore, the integration of CE with cellulose is an attractive strategy, as the cellulose matrix can provide rigid planar supports and binding sites for CE molecules, thereby maintaining the structural properties of the CE while providing a unique surface structure and a stable structure for the guest ions. Bamboo pulp, which is rich in cellulose, has gradually entered the field of heavy metal adsorption research as an environmentally friendly material due to its low cost and green sustainability [18,19]. And aerogels are widely used to remove and recover heavy metals from different aqueous media due to their special nanostructures, with ultra-light weight and high porosity physical properties [20,21].

Microwave radiation-induced chemical reaction technology is a new technology used for polymer synthesis. Compared with traditional heating methods, microwave radiation has the advantages of strong penetration, rapid heating, uniform heating, high efficiency and cleanliness [22]. The application of microwave radiation in cellulose grafting modification can not only make the reaction proceed at a lower temperature, but also improve the activity of the catalyst and monomer while increasing the reaction rate, achieving the purpose of reducing the amount of catalyst and reaction temperature [23].

In this paper, bamboo pulp was used as raw material, and [2,4]-dibenzo-18-crown-6 (DB18C6) was used as a chelating agent. The DB18C6 was anchored on the surface of bamboo pulp aerogel by microwave irradiation. The DB18C6-modified bamboo pulp aerogel (DB18C6/PA) was prepared by directional freeze-drying. The physical and chemical properties of the materials were analyzed by FTIR, XPS, SEM, TEM, TGA, surface area and porosity analyzers. The heavy metals Pb^2+^, Cu^2+^ and Cd^2+^ as adsorbates, the effects of DB18C6 dosage, pH, initial concentration and adsorption temperature on the adsorption properties of Pb^2+^, Cu^2+^ and Cd^2+^ were investigated. At the same time, the adsorption mechanism of DB18C6/PA on heavy metals was explored.

## 2. Results and Discussion

### 2.1. Characterization of MCA

**Figure 1:**
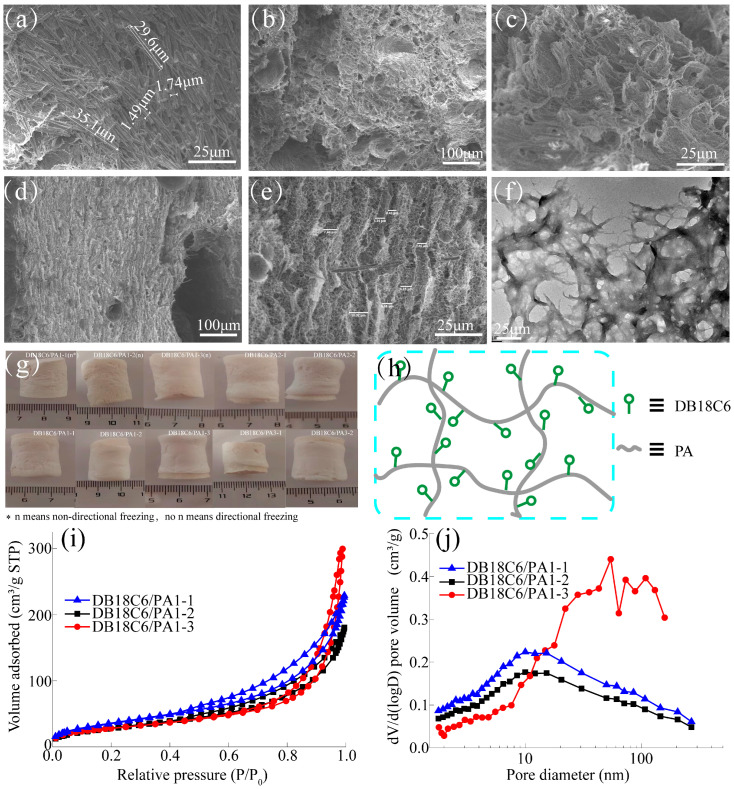
(**a**) SEM images of DB18C6; (**b**,**c**) SEM spectra of DB18C6/PA1-2 obtained by non-directional freezing with different magnification; (**d**,**e**) SEM spectra of DB18C6/PA1-2 obtained by directional freezing with different magnifications; (**f**) TEM of DB18C6/PA1-2 obtained by directional freezing; (**g**) Digital photos of bamboo pulp aerogel obtained under different preparation conditions; (**h**) Schematic diagram of DB18C6/PA structure; (**i**) N_2_ adsorption–desorption isotherm curve of DB18C6/PA; (**j**) Pore size distribution of DB18C6/PA.

Figure 1 shows the SEM, TEM and pore structure of DB18C6, isotropic DB18C6/PA, and anisotropic DB18C6/PA. According to Figure 1a, DB18C6 appeared as slender fibers under the SEM, with a length of about 30 μm and a width of about 1.5 μm. The surface morphology of the isotropic DB18C6/PA is shown in Figure 1b–c, which presents a 3D mesh structure with fibrous DB18C6 attached to the surface [24]. Compared with Figure 1c, Figure 1e shows that DB18C6/PA1-2, prepared using directional freezing technology, has anisotropic channels, indicating that the addition of DB18C6 did not damage the anisotropic structure of the bamboo pulp aerogel. In addition, seven different positions were randomly selected for directional pore analysis, and it was found that the pore size range was 4.46–10.32 μm. Figure 1f shows the TEM of DB18C6/PA1-2, and the material appears homogeneous, without any layering or aggregation caused by additives, indicating good dispersion during the dissolution process. Figure 1g shows that DB18C6 was obtained under different preparation conditions. By comparing and changing the synthesis conditions, such as the addition amount, microwave power, freezing technology and microwave time, the cylindrical aerogels could be successfully synthesized, and the diameter difference was small, indicating that the macro-morphology of the main aerogels was mainly caused by the mold size. In summary, DB18C6 was used as a chelating agent, and it was anchored to the bamboo pulp aerogel network structure by microwave irradiation, as shown in Figure 1g,h. The amount of DB18C6 added had a great influence on the pore structure of the aerogel. Therefore, the pore structure of DB18C6/PA was analyzed at both the micro and macro levels, respectively, and the results are shown in Figure 1i,j and Table 1. Figure 1i shows the N_2_ adsorption–desorption isothermal curves of DB18C6/PA with different addition amounts at 77 K. The results showed that at lower relative pressures, the adsorbed volume of N_2_ increased slowly with increasing relative pressures, indicating that although DB18C6/PA had some micropores, the total volume of the micropores was relatively small. When P/P_0_ was greater than 0.8, the adsorbed volume of nitrogen increased rapidly. According to the IUPAC classification standard, the adsorption–desorption isothermal curve of DB18C6/PA belonged to type IV [25,26], indicating that both mesoporous and macroporous structures existed in the material, which was conducive to the adsorption of heavy metals by DB18C6/PA and the rapid diffusion of the solution in the network structure. Figure 1j shows the pore size distribution of DB18C6/PA with different amounts of DB18C6. The relevant data were fitted by the NLDFT model. It could be seen that with the increase in the DB18C6 addition amount, the total pore volume showed a trend of first decreasing and then increasing. The main reason for this phenomenon was that DB18C6 would block the internal channels of bamboo pulp aerogels; therefore, the total pore volume showed a decreasing trend at first. The total pore volume decreased from 0.3339 cm^3^/g to 0.2633 cm^3^/g. With the increase in the DB18C6 addition, the internal pore wall structure would be further damaged; meanwhile, the pore channels were further connected, and the total pore volume showed an increasing trend. The total pore volume increased from 0.2633 cm^3^/g to 0.4605 cm^3^/g, and the average pore size increased from 10.1555 nm to 18.0939 nm. It could be seen from Table S1 that with the increase in the DB18C6 addition, the porosity decreased from 98.12% to 97.35%, and the specific surface area decreased from 131.51 m^2^/g to 102.46 m^2^/g. This phenomenon was mainly due to the increase in material mass per unit volume caused by the addition of DB18C6.

**Figure 2:**
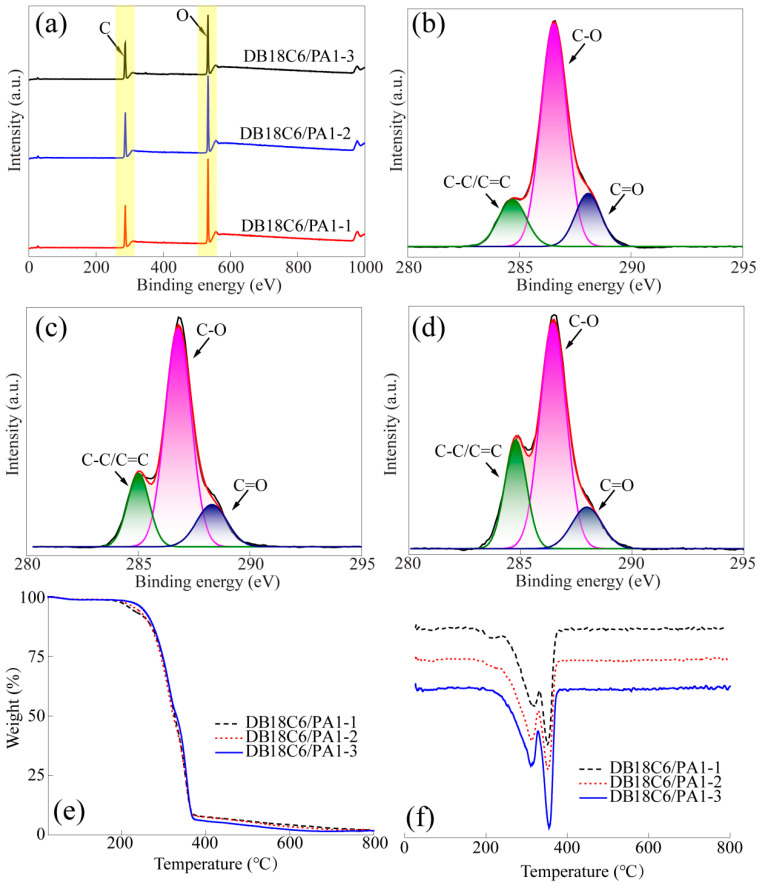
XPS spectra of DB18C6/PA: (**a**) full spectrum, (**b**) C1s spectrum of DB18C6/PA1-1, (**c**) C1s spectrum of DB18C6/PA1-2, (**d**) C1s spectrum of DB18C6/PA1-3; (**e**) TGA and (**f**) DTG of DB18C6/PA.

Figure 2 shows the XPS analysis and thermal stability analysis of DB18C6/PA. Figure 2a shows that the energy spectrum of DB18C6/PA had two characteristic peaks located near 284 eV and 552 eV, representing C1s and O1s, respectively [27]. With the increasing amount of DB18C6 added, the relative values of carbon and oxygen were decreasing, showing a good correlation and indicating that DB18C6 was successfully incorporated into the bamboo pulp aerogel structure. In order to clearly understand the surface chemical structure of DB18C6/PA, the C1s spectrum of DB18C6/PA was analyzed. After the data processing of peak fitting, Figure 2b–d was shown, the results showed that the characteristic peaks located at 284.8, 286.3 and 288.74 eV represented C-C/C=C, C-O and C=O, respectively, which were consistent with the literature reports [28,29,30]. In addition, the peak located near 284.8 eV was significantly enhanced with the increase in the DB18C6 addition during the preparation of the materials, which was mainly caused by the C=C conjugate structure on the benzene ring structure of DB18C6 [31]. Therefore, it was further confirmed that DB18C6 was successfully grafted into the bamboo pulp aerogel. In order to explore the effect of DB18C6 on the thermal stability of the bamboo pulp aerogel, the TGA and DTG results of DB18C6/PA are shown in Figure 2e and Figure 2f, respectively. It can be observed from Figure 2e that the pyrolysis process of DB18C6/PA can be approximately divided into the following three stages: The first stage was from 25 °C to 120 °C, and the mass loss was about 2% in this stage, which was mainly caused by the evaporation of free water in the materials [32]. The second stage was from 120 °C to 370 °C, and the mass loss was about 93%. This stage was mainly caused by the formation of a large number of small molecular gasses, which was caused by the fracture of an alcohol hydroxyl group, carbon-carbon bond and carbon-oxygen bond in DB18C6/PA [33,34]. The third stage was from 370 °C to 800 °C, the mass loss was approximately 3%. The main reason was the residue after carbonization of DB18C6/PA, which gradually formed a graphite structure through the aromatic cyclization process [35]. In order to explore the process of the main degradation stage (the second stage), the TGA curve of DB18C6/PA was derived, and the DTG curve is shown in Figure 2f. The results showed that there were two maximum values at 311 °C and 355 °C between 120 °C and 370 °C, which were mainly caused by the formation of new chemical components after DB18C6 was successfully grafted into the bamboo aerogels.

### 2.2. Adsorption Studies of Pb^2+^, Cu^2+^ and Cd^2+^

#### 2.2.1. Effects of Preparation and Adsorption Conditions on Adsorption Performance

**Figure 3:**
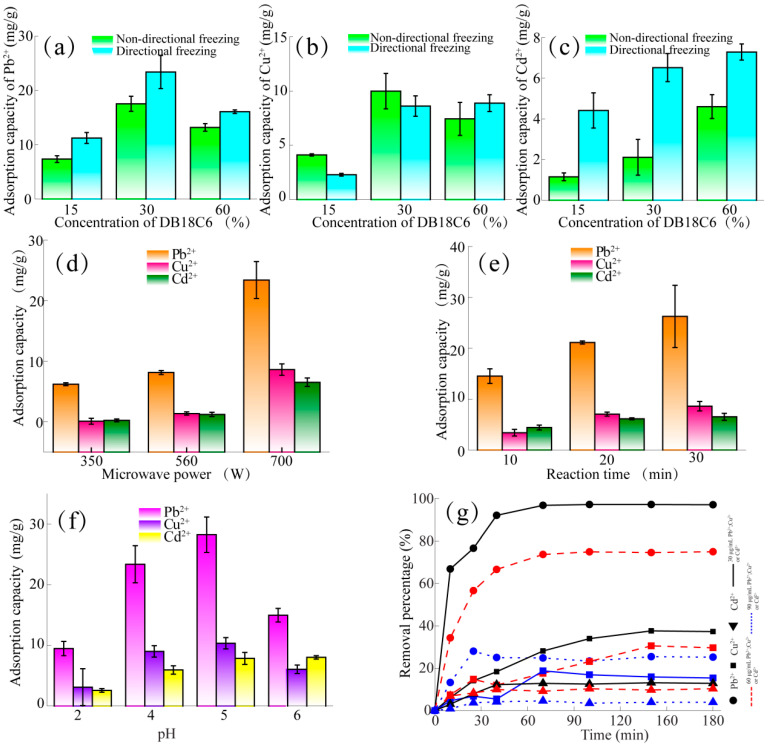
Effect of DB18C6/PA obtained by non-directional freezing and directional freezing on adsorption of (**a**) Pb^2+^, (**b**) Cu^2+^, and (**c**) Cd^2+^. Effect of DB18C6/PA obtained with different (**d**) microwave power and (**e**) reaction time on the adsorption of Pb^2+^, Cu^2+^ and Cd^2+^. The effect of (**f**) pH and (**g**) initial concentration on the adsorption of Pb^2+^, Cu^2+^ and Cd^2+^ by DB18C6/PA.

The adsorption of Pb^2+^, Cu^2+^ and Cd^2+^ was explored under different preparation conditions, such as DB18C6 addition amount, freezing technology, microwave power and microwave time. Firstly, DB18C6/PA1-1, DB18C6/PA1-2 and DB18C6/PA1-3 prepared by directional freezing and non-directional freezing were used to test Pb^2+^, Cu^2+^ and Cd^2+^ adsorption, respectively, and the results are shown in Figure 3a–c. In general, with the increase in the DB18C6 addition, the adsorption capacity of Pb^2+^, Cu^2+^ and Cd^2+^ of DB18C6/PA, prepared by directional freezing or non-directional freezing, showed a trend of first increasing and then decreasing or stabilizing. Due to the addition of DB18C6, some pores were blocked, and the specific surface area of the material decreased slightly, but the adsorption site was increased within a certain range [36]. Therefore, the adsorption capacity showed an increasing trend at first. However, when the addition of DB18C6 was further increased, the internal pore wall of the material collapsed, and the pore volume increased, with the adsorption capacity showing a decreasing or stable trend. As shown in Figure 3a, compared with the isotropic DB18C6/PA, the anisotropic DB18C6/PA showed a higher adsorption capacity for Pb^2+^ in the same adsorption condition. When the amount of DB18C6 was 30%, the adsorption capacity of Pb^2+^ was 23.39 mg/g, which was the highest value. Figure 3b shows that the adsorption capacity of Cu^2+^ by isotropic or anisotropic DB18C6/PA has no significant rule in the same adsorption condition. However, with the increase in the DB18C6 addition, the adsorption capacity of Cu^2+^ showed a trend of first increasing and then decreasing. Similarly, when the DB18C6 addition was 30%, the adsorption capacity of Cu^2+^ reached a peak at 10.00 mg/g.

As shown in Figure 3c, in the same formulation, compared with the isotropic DB18C6/PA, the anisotropic DB18C6/PA also presented a higher adsorption capacity for Cd^2+^, and with the increase in the added amount of DB18C6, the adsorption capacity of Cd^2+^ presented a tendency to rise at first, and when the added amount of DB18C6 was 60%, the adsorption capacity of Cd^2+^ reached a peak at 7.29 mg/g. The DB18C6/PA adsorption effect of Pb^2+^, Cu^2+^ and Cd^2+^ by freezing technology and addition amount was comprehensively considered, and the 30% DB18C6 addition amount was used for subsequent research.

Secondly, cerium ammonium nitrate had the advantages of low activation energy, mild reaction conditions, and fast initiation speed [37]. As initiator, Ce^4+^ chelated with the two adjacent hydroxyl groups of C2 and C3 on the glucose unit of the bamboo pulp cellulose. With the reduction of Ce^4+^ to Ce^3+^, the bond between C2 and C3 was broken, at the same time, free radicals were generated on the cellulose backbone. Then, the free radicals on the backbone reacted with the benzene ring in DB18C6, and DB18C6 was grafted onto the bamboo pulp aerogel. Especially when materials were heated via microwave irradiation, the high-frequency reciprocating motion of internal dipole molecules further enhanced the process, making the reaction easier. Therefore, the effects of DB18C6/PA2-1, DB18C6/PA2-2 and DB18C6/PA1-2, prepared by different microwave power, on the adsorption capacities of Pb^2+^, Cu^2+^ and Cd^2+^ were explored, and the results are shown in Figure 3d. With the increase in microwave power, the adsorption capacities of Pb^2+^, Cu^2+^ and Cd^2+^ showed a gradually increasing trend [38]. When the microwave power reached 700 W, the adsorption capacities of Pb^2+^, Cu^2+^ and Cd^2+^ by DB18C6/PA reached their maximum values, which were 23.39 mg/g, 8.61 mg/g and 6.52 mg/g, respectively.

Finally, because the time of microwave heating during the reaction affects the process of free radical reaction, it simultaneously affected the adsorption amount of Pb^2+^, Cu^2+^ and Cd^2+^ by DB18C6/PA. Therefore, the effects of DB18C6/PA3-1, DB18C6/PA3-2 and DB18C6/PA1-2 prepared at different reaction times on the adsorption capacities of Pb^2+^, Cu^2+^ and Cd^2+^ were explored, and the results are shown in Figure 3e. With the extension of reaction time, the adsorption capacity of Pb^2+^, Cu^2+^ and Cd^2+^ showed a trend of first increasing and then stabilizing. When the reaction time was 30 min, the adsorption capacities of Pb^2+^, Cu^2+^ and Cd^2+^ by DB18C6/PA reached their maximum values. In summary, the synthesis conditions of anisotropic DB18C6/PA1-2 were used for the subsequent study.

Figure 3f shows that DB18C6/PA had a significant effect on the adsorption capacity of Pb^2+^, Cu^2+^ and Cd^2+^ at different pH values. With the increase in pH value, it showed a trend of first increasing and then decreasing. At a low pH, DB18C6/PA showed a low adsorption of Pb^2+^, Cu^2+^ and Cd^2+^. Due to the protonation of the material surface under the low pH, it had the same charge with the surface of the adsorbed heavy metals, resulting in a repulsive force that caused a longer distance between the adsorbent and the adsorbate [39]. Therefore, the adsorption capacity of the adsorbent decreased. With the increase in the pH value in the solution, the negative charge on the surface of DB18C6/PA increased, so the adsorption capacity of Pb^2+^, Cu^2+^ and Cd^2+^ increased rapidly. When pH > 5.0, Pb^2+^, Cu^2+^ and Cd^2+^ existed as hydrated ions, so the adsorption capacity of the adsorbent decreased. Thus, when pH = 5, the adsorption capacities of Pb^2+^, Cu^2+^ and Cd^2+^ were the highest, with values of 26.11 mg/g, 9.59 mg/g and 7.29 mg/g, respectively. The results showed that DB18C6/PA had a high ability to capture three heavy metal ions, especially Pb^2+^.

In addition to pH, the initial concentration was one of the other key parameters affecting the adsorption process. Therefore, the removal rates of 30–90 μg/mL Pb^2+^, Cu^2+^ and Cd^2+^ were tested by a different DB18C6/PA at temperature = 25 °C and pH = 4; the results are shown in Figure 3g. In the single system of Pb^2+^, Cu^2+^ and Cd^2+^, the removal efficiency decreased with the increase in heavy metal concentrations. This phenomenon was mainly caused by the increasing driving force of adsorbate diffusion to the surface-active sites of the adsorbent, which gradually occupied the surface-active sites of the adsorbent [40]. The dissociation degree of DB18C6/PA surface functional groups was reduced, as well as the hindering effect of excess ions in the solution during the adsorption process. In addition, the results showed that contact time affects the adsorption performance of the adsorbent DB18C6/PA, indicating that a large number of active sites of the adsorbent rapidly combined with the adsorbate at the beginning of the adsorption, As the adsorption time progresses, the number of active sites decreased, and then the adsorption equilibrium was reached. Therefore, the adsorption time of 180 min was selected for subsequent experiments to ensure that the adsorbate reached adsorption saturation.

#### 2.2.2. Adsorption Kinetics, Adsorption Isothermal Curve and Thermodynamic Parameters Analysis

**Figure 4:**
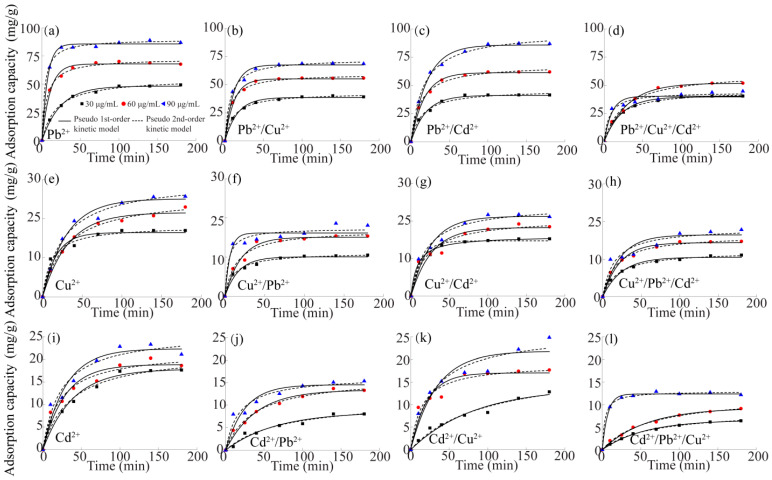
The fitted curves of adsorption kinetics models for Pb^2+^ in the single, binary and ternary systems of (**a**) Pb^2+^, (**b**) Pb^2+^/Cu^2+^, (**c**) Pb^2+^/Cd^2+^, (**d**) Pb^2+^/Cu^2+^/Cd^2+^; Cu^2+^ in the single, binary and ternary systems of (**e**) Cu^2+^, (**f**) Cu^2+^/Pb^2+^, (**g**) Cu^2+^/Cd^2+^, (**h**) Cu^2+^/Pb^2+^/Cd^2+^; Cd^2+^ in the single, binary and ternary system of (**i**) Cd^2+^, (**j**) Cd^2+^/Pb^2+^, (**k**) Cd^2+^/Cu^2+^, (**l**) Cd^2+^/Pb^2+^/Cu^2+^, on the DB18C6/PA, at pH = 5 and temperature = 25 °C.

In order to illustrate the control steps of the adsorption rate and absorption types, the adsorption kinetics data were fitted using the pseudo-first-order kinetic model (Equation (S1)) and pseudo-second-order kinetic model (Equation (S2)). Figure 4 shows the adsorption kinetics of DB18C6/PA on single, binary and ternary systems of Pb^2+^, Cu^2+^ and Cd^2+^ at different concentrations (30–90 μg/mL). The results showed that the adsorption capacity of Pb^2+^, Cu^2+^ and Cd^2+^ increased with an increase in the heavy metal concentration. Mainly, the high concentration of Pb^2+^, Cu^2+^ and Cd^2+^ solutions would provide a greater possibility of capturing DB18C6/PA, which was also consistent with previous reports [41,42,43,44]. In addition, at the beginning, the adsorption process showed that the adsorbates (Pb^2+^, Cu^2+^ and Cd^2+^) were adsorbed rapidly, and then the adsorption rate decreased until it tended to be stable. In particular, the adsorption rate of the high concentration system was faster than that of the low concentration system. The reason for this phenomenon was that there was a large concentration difference between the adsorbate in DB18C6/PA and the solution at the beginning of the adsorption. The large concentration difference led to a large mass transfer driving force; therefore, the diffusion rate was accelerated [45,46]. As time went by, the concentration difference in heavy metals between DB18C6/PA and the solution gradually decreased; meanwhile, the number of active sites gradually decreased until the adsorption equilibrium was reached. The adsorption capacity of DB18C6/PA in the multivariate system was lower than that in the single system because the different heavy metals, Pb^2+^, Cu^2+^ and Cd^2+^, competed with each other at the same adsorption site in the multivariate system. The relevant data from DB18C6/PA in the adsorption process of Pb^2+^, Cu^2+^ and Cd^2+^ were fitted, and the results are shown in Tables S2–S4. The fitting results of the pseudo-second-order kinetic model were better than that of the pseudo-first-order kinetic model, indicating that chemical adsorption was also involved in the adsorption process [47].

**Figure 5:**
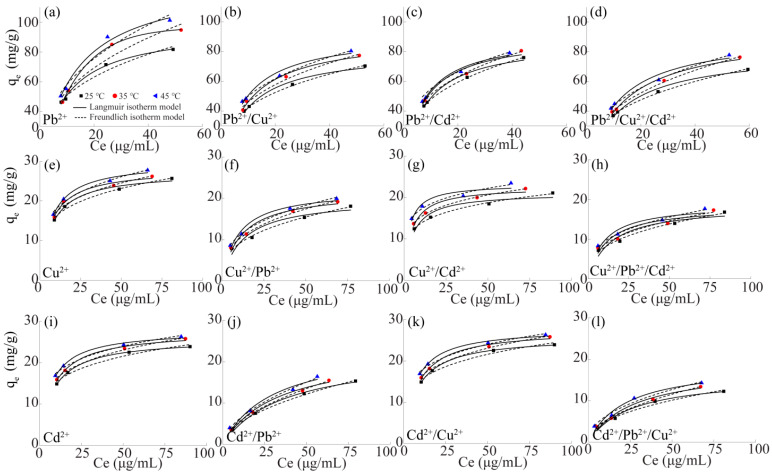
The fitted curves of adsorption isothermal models for Pb^2+^ in the single, binary and ternary systems of (**a**) Pb^2+^, (**b**) Pb^2+^/Cu^2+^, (**c**) Pb^2+^/Cd^2+^, (**d**) Pb^2+^/Cu^2+^/Cd^2+^; Cu^2+^ in the single, binary and ternary systems of (**e**) Cu^2+^, (**f**) Cu^2+^/Pb^2+^, (**g**) Cu^2+^/Cd^2+^, (**h**) Cu^2+^/Pb^2+^/Cd^2+^; Cd^2+^ in the single, binary and ternary system of (**i**) Cd^2+^, (**j**) Cd^2+^/Pb^2+^, (**k**) Cd^2+^/Cu^2+^, (**l**) Cd^2+^/Pb^2+^/Cu^2+^ on the DB18C6/PA.

In order to accurately describe the relationship between the concentrations of Pb^2+^, Cu^2+^ and Cd^2+^ in DB18C6/PA and those in the solution at a certain temperature, the Langmuir adsorption isothermal model (Equation (S3)) and Freundlich adsorption isothermal model (Equation (S4)) were used to fit the relevant data. Figure 5 shows the adsorption isothermal models for Pb^2+^, Cu^2+^ and Cd^2+^ in the single and multivariate systems were studied at different temperatures. The results showed that the adsorption capacity of Pb^2+^, Cu^2+^ and Cd^2+^ into DB18C6/PA increased with increasing equilibrium concentration at different temperatures. At the same time, the adsorption capacity of Pb^2+^, Cu^2+^ and Cd^2+^ adsorbed by DB18C6/PA increased with the increase in temperature, indicating that the adsorption process was endothermic and the increase in temperature was conducive to the adsorption. The Langmuir and Freundlich isotherm models were used to fit the relevant data, and the parameters of the adsorption isotherm curves in single, binary and ternary systems are shown in Tables S5–S7. By comparing the correlation coefficients, the results were as follows: (i) The Langmuir isotherm model could accurately describe the adsorption of Pb^2+^ by DB18C6/PA, indicating that the adsorption process of Pb^2+^ by DB18C6/PA was mainly chemisorption [48]. The adsorption isothermal curves of Pb^2+^ in multivariate systems could be described by the Freundlich adsorption isothermal model, indicating that the adsorption process was a multi-molecular layer adsorption, and electrostatic interaction, ion exchange, etc., existed between adsorbents and adsorbents [49]. (ii) Most of the adsorption isothermal curves for Cu^2+^ and Cd^2+^ in the single and multivariate systems could be described by the Freundlich adsorption isothermal model, which indicated that the process was a multi-molecular layer adsorption and proved the existence of physical adsorption. (iii) When the temperature was 45 °C, the adsorption capacity of Pb^2+^ reached the maximum in the systems of Pb^2+^, Pb^2+^/Cu^2+^, Pb^2+^/Cd^2+^ and Pb^2+^/Cu^2+^/Cd^2+^, and the maximum adsorption capacities were 129.15, 92.30, 91.36 and 91.72 mg/g, respectively. The maximum adsorption capacity of Cu^2+^ in the systems of Cu^2+^, Cu^2+^/Pb^2+^, Cu^2+^/Cd^2+^ and Cu^2+^/Pb^2+^/Cd^2+^ were 29.85, 22.14, 23.22 and 18.83 mg/g, respectively. The maximum adsorption capacity of Cd^2+^ in the systems of Cd^2+^, Cd^2+^/Pb^2+^, Cd^2+^/Cu^2+^ and Cd^2+^/Pb^2+^/Cu^2+^ were 27.89, 24.14, 26.68 and 18.92 mg/g, respectively.

In order to describe the energy change in the adsorption process more clearly, the thermodynamic parameters of the adsorption process were calculated using Equations (S5)–(S8). Tables S8–S10 shows the thermodynamic parameters of DB18C6/PA for the adsorption of Pb^2+^, Cu^2+^ and Cd^2+^ in the single, binary and ternary systems. The values of ∆Gθ were all negative, indicating that the adsorption process of DB18C6/PA for Pb^2+^, Cu^2+^ and Cd^2+^ was a spontaneous process. ∆H^θ^ > 0, which reflected the endothermic nature of the adsorption process. ∆S^θ^ > 0 represented an increase in the degrees of freedom at the solid–liquid interface during the adsorption process [50].

### 2.3. Adsorption Selectivity

In order to explore the adsorption selectivity of Pb^2+^, Cu^2+^ and Cd^2+^ by DB18C6/PA in the multivariate system, Pb^2+^, Cu^2+^ and Cd^2+^ solutions with a concentration of 30 μg/mL were selected to be evaluated, and the distribution coefficient (K_d_) [51] and separation factors (α_M_^A^) [52] were used as evaluation indexes. K_d_ represented the strength of the affinity between the adsorbent and the adsorbate, L/g, the calculation formula is shown in Equation (S9); α_M_^A^ represented the adsorption selectivity coefficient of the adsorbent for the A ion in a solution with interfering ions (M) and the calculation formula is shown in Equation (S10). Table S11 shows the distribution coefficients and separation factors of DB18C6/PA for Pb^2+^, Cu^2+^ and Cd^2+^ in single, binary and ternary systems. By comparing the distribution coefficients, the adsorption selectivity of DB18C6/PA for the three heavy metals was ranked in the following order: Pb^2+^ >> Cu^2+^ > Cd^2+^. In addition, when Pb^2+^ coexists with Cu^2+^ and Cd^2+^, the K_d_ value decreases, indicating that Pb^2+^, Cu^2+^ and Cd^2+^ are competitive in the adsorption process. By comparing the separation factors (α_M_^A^), the same results could be obtained by ordering the adsorption selectivity of DB18C6/PA for the three heavy metals. At the same time, DB18C6/PA had a stronger adsorption selectivity of Pb^2+^ than Cu^2+^ and Cd^2+^ in the multivariate system. When only Cu^2+^ and Cd^2+^ exist in the system, DB18C6/PA has a stronger adsorption selectivity for Cu^2+^.

### 2.4. Adsorption Mechanism

**Figure 6:**
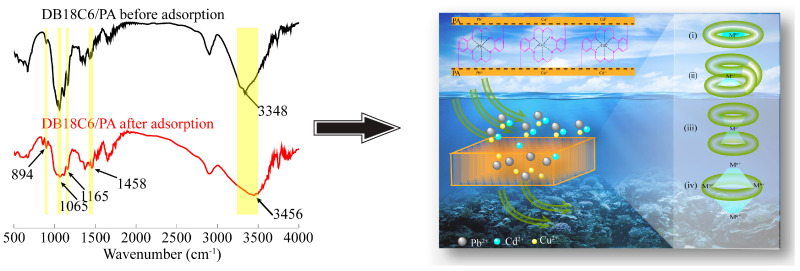
Adsorption mechanism of DB18C6/PA on Pb^2+^, Cu^2+^ and Cd^2+^.

By studying the adsorption kinetics model, adsorption isotherm curve model, and thermodynamic parameters of DB18C6/PA during the adsorption process, it was determined that there were multiple adsorption processes involved in the adsorption of Pb^2+^, Cu^2+^ and Cd^2+^, including physical adsorption and chemical adsorption. In order to explore the surface chemical structure changes in DB18C6/PA in the adsorption process, the DB18C6/PA before and after adsorption were characterized by FTIR, and the results are shown in Figure 6. The characteristic absorption peaks of DB18C6/PA before adsorption were as follows: the wide and strong peak of 3348 cm^−1^ represented the O-H stretching vibration peak of association; 1458 cm^−1^ represented the stretching vibration of C=C bonds; and the peaks of 1165, 1065 and 894 cm^−1^ represented C-O stretching vibration [53,54]. The above results were consistent with the functional group structures of the hydroxyl group in cellulose, benzene ring in DB18C6, and ether bond in crown ether and cellulose, which indicated the successful synthesis of DB18C6/PA.

Compared with DB18C6/PA before adsorption, the position or shape of the characteristic absorption peak changes after adsorption, and the stretching vibration peak of O-H becomes wider and the position changes to 3456 cm^−1^, indicating that the bamboo pulp matrix has active sites for Pb^2+^, Cu^2+^ and Cd^2+^ adsorption. The peak intensity of the C-O contraction vibration was weakened and slightly changed, which was caused by Pb^2+^, Cu^2+^ and Cd^2+^ being captured by the crown ether ring in DB18C6/PA and forming a new coordination bond binding. In the process of capture, the stability of DB18C6/PA binding to Pb^2+^, Cu^2+^ and Cd^2+^ mainly depended on the relative size of the crown ether hollow ring and heavy metal ions. Generally, the combination types [55] are presented as (i)–(iv), as shown in Figure 6. When the size of the crown ether ring was similar to the metal ion, a size-matched complex (e.g., i) or an encapsulated complex (e.g., ii) were formed, these complexes of 1:1 (n_metal ion_:n_crown ether_) were generally relatively stable. When there was a size difference between the metal ion and the crown ether ring, a sandwich complex of 2:1 (e.g., iii) or a binuclear complex of 1:2 (e.g., iv) were formed; in general, these were relatively unstable. Therefore, Pb^2+^ and oxygen atoms of DB18C6 might exist mainly to form size-matching complexes (e.g., i) or encapsulated complexes (e.g., ii), while copper and cadmium were mainly captured in the form of sandwich complexes (e.g., iii) or binuclear complexes (e.g., iv).

### 2.5. Desorption and Regeneration

**Figure 7:**
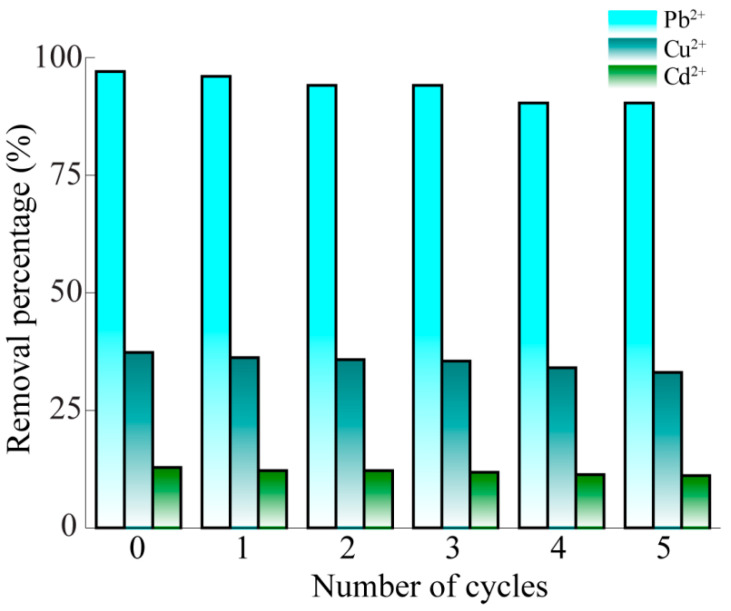
Removal efficiency of Pb^2+^, Cu^2+^ and Cd^2+^ by DB18C6/PA for 5 adsorption–desorption cycles.

In the practical application process, the stability and regeneration of the adsorbent were important factors. DB18C6/PA had a high adsorption capacity at pH = 5.0, and it was not conducive to the adsorption of Pb^2+^, Cu^2+^ and Cd^2+^ at low pH levels. At a low pH, there is a fairly high concentration of hydrogen ions around the binding site of the adsorbent, which strongly competes with the positively charged heavy metal ions [56]. The surface of the adsorbent material is protonated and has the same charge as the surface of the adsorbed heavy metal, resulting in the strong electrostatic repulsion of metal cations and a longer distance between the adsorbent and the adsorbate [57]. Therefore, 0.1 mol/L hydrochloric acid solution was used to desorb the Pb^2+^, Cu^2+^ and Cd^2+^ in DB18C6/PA, and the results are shown in Figure 7. Compared with the first time, after 5 times of elution and regeneration, the removal efficiency of Pb^2+^, Cu^2+^ and Cd^2+^ by DB18C6/PA was decreased by 6.86%, 11.47% and 13.31%, respectively. It can be seen that DB18C6/PA has good reusability and it can effectively reduce the amount of solid waste in practical applications. For comparison, we retrieved the metal ion adsorption capacity of other crown ether-based/functionalized adsorbents and systematically listed the adsorbents, adsorbates and the adsorption capacity. As shown in Table S12, in our work, an anisotropic crown ether-grafted bamboo pulp aerogel was used as a chelating agent for the selective adsorption of heavy metal Pb^2+^ with a maximum adsorption capacity of 129.15 mg/g, which was better than the related research progress [58,59,60,61]. This has a specific application potential in water pollution control.

## 3. Conclusions

In this paper, DB18C6-modified bamboo pulp hydrogel was synthesized by microwave irradiation using bamboo pulp as the raw material and dibenzo-18-crown-6 as the capture reagent. Anisotropic DB18C6/PA was prepared by a directional freeze-drying method; at the same time, the morphology and chemical structure were characterized by XPS, SEM, TEM, TGA and BET. The effects of the DB18C6 addition amount, pH, initial concentration and adsorption temperature on the adsorption capacity of Pb^2+^, Cu^2+^ and Cd^2+^ were investigated. DB18C6/PA had many characteristics such as a 3D directional pore, high porosity (97.67%), large specific surface area (103.7 m^2^/g) and good thermal stability. The surface had various functional groups such as hydroxyl group, aldehyde group and ether bond. The adsorption of Pb^2+^, Cu^2+^ and Cd^2+^ by DB18C6/PA in single and multivariate systems conformed to the pseudo-second-order kinetic model, while most of which conformed to the Freundlich adsorption isothermal model, and a few conformed to the Langmuir adsorption isothermal model, indicating that the adsorption process involved both chemical adsorption and physical adsorption, and the adsorption process was a spontaneous endothermic process. In the single system, the maximum adsorption capacities of Pb^2+^, Cu^2+^ and Cd^2+^ by DB18C6/PA were 129.15, 29.85 and 27.89 mg/g, respectively. The adsorption selectivity of DB18C6/PA for Pb^2+^, Cu^2+^ and Cd^2+^ in the adsorption process of multivariate systems was ranked in the following order: Pb^2+^ >> Cu^2+^ > Cd^2+^; moreover, there was competitive adsorption between Pb^2+^, Cu^2+^ and Cd^2+^ in the adsorption process.

## 4. Materials and Methods

### 4.1. Chemicals and Materials

Bamboo pulp was purchased from Sichuan Tianzhu Bamboo Resources Development Co., Ltd., Yibin, China. DB18C6 (AR, purity ≥99%) was purchased from Shanghai Macklin Biochemical Technology Co., Ltd., Shanghai, China. Cerium ammonium nitrate (AR, purity ≥ 99%), Pb(NO_3_)_2_ (AR, purity ≥ 99%), Cu(NO_3_)_2_ (AR, purity ≥ 99%) and Cd(NO_3_)_2_ (AR, purity ≥ 99%) were purchased from West Asia Chemical Technology (Linyi, Shandong, China) Co., Ltd.

### 4.2. Preparation of DB18C6/PA

Firstly, 0.0465 g, 0.093 g and 0.186 g DB18C6 powder were weighed into three 50 mL beakers in turn, and 20 g NMMO and 3.04 mL pure water were added in turn. The mixture was completely scattered at 90 °C. An amount of 0.31 g of bamboo pulp was added to the mixture, stirred for 120 min, poured into molds to cool, and the formed gel was left at room temperature, aged overnight and soaked in deionized water to replace the NMMO in the gel. The bamboo pulp hydrogels with different DB8C6 additions were prepared (among the three hydrogels, the mass of DB8C6 accounted for 15%, 30% and 60% of the total mass of bamboo). Then, the cellulose hydrogel was immersed in 30 mL 0.02 mol/L ceric ammonium nitrate aqueous solution for 8 h, and the grafting reaction was carried out in a microwave generator at 700 W with a reaction time of 30 min. Finally, the unreacted DB18C6 was washed out by soaking in methanol, and the methanol in the gel was repeatedly soaked and replaced with 20% tert butanol water solution. The sample DB18C6/PA was obtained by pre-freezing in liquid nitrogen at -196 °C and freeze-drying for 48 h. Samples DB18C6/PA1-1, DB18C6/PA1-2 and DB18C6/PA1-3 represented the additives of 15%, 30% and 60%, respectively.

An amount of 0.093 g DB8C6 powder additive was accurately weighed and synthesized as described above. Samples DB18C6/PA2-1 and DB18C6/PA2-2 represented a microwave power of 350 W and 560 W, respectively, with the reaction time of 30 min. Samples DB18C6/PA3-1 and DB18C6/PA3-2 represented reaction times of 10 min and 20 min, respectively, with the microwave power of 700 W. The synthesis method and reagent dosage are shown in Table 1. The directional freezing mold was a self-made mold, which mainly included a cylindrical mold and an I-shaped heat transfer copper rod. The schematic diagram of the synthesis process is shown in Figure S1.

### 4.3. Characterization

The morphology of aerogel was characterized by SEM (Regulus 8100, Hitachi, Tokyo, Japan) and TEM (Tecnai G2 F20, FEI, Hillsboro, OR, USA). The chemical compositions were characterized by XPS (Escalab 250Xi, ThermoFisher, Waltham, MA, USA). Pore structure was analyzed by specific surface area and pore analyzer (ASAP 2020, Micromeritics, Atlanta, GA, USA). Thermal properties analysis was conducted using thermogravimetric analysis (TGA, STA 449C, NETZSCH, Selb, Germany). Infrared spectroscopy analysis (FTIR) was performed using KBr pellets method and an IRAffinity-1S Fourier transform infrared spectrometer from Shimadzu, Kyoto, Japan. The concentration of lead, copper and cadmium ions in the solution was measured using an atomic absorption spectrometer (AAS, PinAAcle 900T, PerkinElimer, Waltham, MA, USA).

### 4.4. Adsorption Experiment of Pb^2+^, Cu^2+^ and Cd^2+^

The adsorption performance of DB18C6/PA were evaluated using single or multi-component heavy metal solutions (Pb^2+^, Cu^2+^ and Cd^2+^). The solution was adjusted to the desired pH by 0.1 mol/L sodium hydroxide or hydrochloric acid. A certain mass of DB18C6/PA was put into a 150mL conical flask, and 50 mL of heavy metal solution was added. The conical flask was placed in a shaker (WHY-2A, KEXI Instrument, Xiamen, China) and shaken for 180 min at a speed of 120 r/min. At regular intervals, the concentration of heavy metals in the solution before and after adsorption was measured by an atomic absorption spectrometer. In the multi-component solution, the initial concentration of heavy metal ions in each component was the same. Equation (1) was used to calculate the adsorption capacity (*q_t_*), and Equation (2) was used to calculate the removal efficiency (*η*). The experiment was repeated three times in parallel.

(1) $$q_{t}(\mathrm{mg}/g)=\frac{(C_{0}-C_{1})\times V}{m}$$

(2) $$\eta \left(\%\right)=\frac{C_{0}-C_{1}}{C_{0}}\times 100$$

where *C*_0_ is the concentration of heavy metals in the solution before adsorption, mg/mL; *C*_1_ is the concentration of heavy metals in the solution after adsorption, g/mL; *V* is the volume of sample solution, mL; *m* is the weight of the sample, g.

### 4.5. Regenerative Performance

After the adsorption experiment, DC18C6/PA was removed from the solution and regenerated as follows. First, the free Pb^2+^, Cu^2+^ and Cd^2+^ attached to the surface of DC18C6/PA were removed by soaking in deionized water for 2 h. Then, DC18C6/PA was eluted in 0.1 mol/L hydrochloric acid aqueous solution. Finally, it was repeatedly washed with deionized water to neutral and then lyophilized. These processes were repeated five times, and the removal efficiency was used to evaluate this process.

## Figures and Tables

**Table 1:** Synthesis method and reagent dosage.

No.	Additive Dosage/%	Microwave Power/W	Reaction Time/min	Frozen Type
DB18C6/PA1-1	15	700	30	n/d *
DB18C6/PA1-2	30	700	30	n/d
DB18C6/PA1-3	60	700	30	n/d
DB18C6/PA2-1	30	700	10	d
DB18C6/PA2-2	30	700	20	d
DB18C6/PA3-1	30	560	30	d
DB18C6/PA3-2	30	350	30	d

* n means non-directional freezing; d means directional freezing.

## Data Availability

The original contributions presented in this study are included in the article/Supplementary Material. Further inquiries can be directed to the corresponding author(s).

## Supplementary Materials

The following supporting information can be downloaded at: https://www.mdpi.com/article/10.3390/gels10120778/s1, Table S1: Specific surface area, pore volume and porosity of DB18C6/PA with different amounts of modifiers; Table S2: DB18C6/PA adsorption kinetics fitting parameters for Pb^2+^ in single, binary and ternary systems; Table S3: DB18C6/PA adsorption kinetics fitting parameters for Cu^2+^ in single, binary and ternary systems; Table S4: DB18C6/PA adsorption kinetics fitting parameters for Cd^2+^ in single, binary and ternary systems; Table S5: DB18C6/PA adsorption isotherm curve fitting parameters for Pb^2+^ in single, binary and ternary systems; Table S6: DB18C6/PA adsorption isotherm curve fitting parameters for Cu^2+^ in single, binary and ternary systems; Table S7: DB18C6/PA adsorption isotherm curve fitting parameters for Cd^2+^ in single, binary and ternary systems; Table S8: Thermodynamic parameters of Pb^2+^ adsorption by DB18C6/PA in single, binary and ternary systems; Table S9: Thermodynamic parameters of Cu^2+^ adsorption by DB18C6/PA in single, binary and ternary systems; Table S10: Thermodynamic parameters of Cd^2+^ adsorption by DB18C6/PA in single, binary and ternary systems; Table S11: Adsorption distribution coefficient and selectivity factor of DB18C6/PA for Pb^2+^, Cu^2+^ and Cd^2+^ in single, binary and ternary systems. Figure S1: The schematic diagram of the synthesis process.
