# Supplementary material for: Microwave Irradiation-Assisted Synthesis of Anisotropic Crown Ether-Grafted Bamboo Pulp Aerogel as a Chelating Agent for Selective Adsorption of Heavy Metals (Mn+)
Source: Gels. 2024 Nov 28;10(12):778. doi: 10.3390/gels10120778 (PMC11675747; doi:10.3390/gels10120778)
Supplement: Supplementary file 1 [file gels-10-00778-s001.zip › gels-3324807-supplementary.pdf]

Supporting Information

Microwave Irradiation-Assisted Synthesis of an Anisotropic Crown Ether Grafted Bamboo Pulp Aerogel as a Chelating Agent for Selective Adsorption of Heavy Metals (M^{n+})

Wenxiang Jing[a,b,c], Min Tang[b], Xiaoyan Lin[a,c]*, Chai Yang[b], Dongming Lian[b], Ying Yu[b], Dongyang Liu[b]

a. School of Materials and Chemistry, Southwest University of Science and Technology, 621010, Mianyang, Sichuan, China

b. Yibin Forestry and Bamboo Industry Research Institute , Yibin 644005, China.

c. Engineering Research Center of Biomass Materials, Ministry of Education, Southwest University of Science and Technology, Mianyang 621010, China.

** Corresponding author: Xiaoyan Lin; E-mail: lxy20100205@163.com*

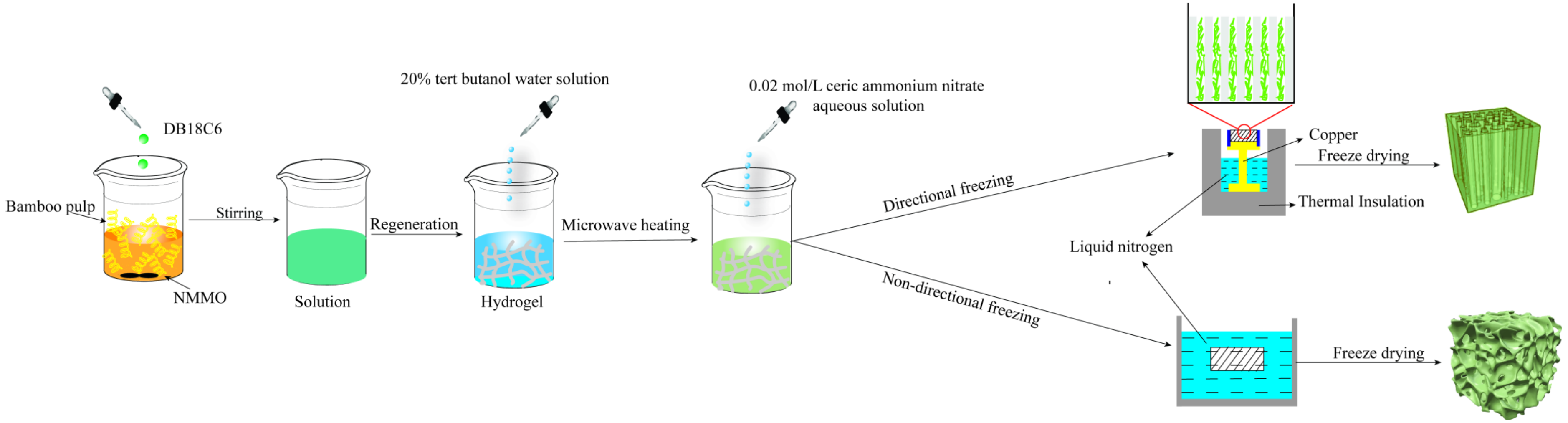

Figure S1 The schematic diagram of the synthesis process.

Table S1 Specific surface area, pore volume and porosity of DB18C6/PA with different amounts of modifiers

No.	S_{BET} /$m^2 \cdot g^{-1}$	D_{pore} /nm	$V_{total\ pore}$ /$cm^3 \cdot g^{-1}$	$V_{<50nm}$ /$cm^3 \cdot g^{-1}$	$V_{<50\ nm}/V_{total\ pore}$	ρ/%
DB18C6/PA1-1	131.51	10.1552	0.3339	0.09171	0.2747	98.12
DB18C6/PA1-2	103.70	10.1555	0.2633	0.07231	0.2746	97.67
DB18C6/PA1-3	102.46	18.0939	0.4605	0.12373	0.2686	97.35

ρ_0 is the density of cellulose，1.6 g/cm^3

Pseudo-first-order kinetic model (Equation S1) and pseudo-second-order kinetic model (Equation S2) are as follows：

$$q_t = q_e(1 - e^{-k_1 t})$$ （**Equation S1**）

$$q_t = k_2 q_e^2 t / (1 + k_2 q_e t)$$ （**Equation S2**）

Where q_e and q_t are the amounts of Pb^{2+}, Cu^{2+}, or Cd^{2+} adsorbed at equilibrium and t min, respectively, mg/g; K_1 is the rate constant of the pseudo-first-order kinetic model, min^{-1}; K_2 is the rate constant of the pseudo-second-order kinetic model, g/(mg·min); t is the adsorption time, min.

Table S2 DB18C6/PA adsorption kinetics fitting parameters for Pb^{2+} in single, binary, and ternary systems

Adsorbates	Concentration (mg/L)	Pseudo-first-order model			Pseudo-second-order model		
		K_1 (min^{-1})	q_e (mg/g)	R^2	K_2 (g/mg·min)	q_e (mg/g)	R^2
Pb^{2+}	30	0.0417	48.23	0.9824	0.000985	55.33	0.9934
	60	0.0947	67.96	0.9883	0.00223	72.81	0.9948
	90	0.1406	85.75	0.9949	0.00320	89.85	0.9954
Pb^{2+}/Cu^{2+}	30	0.0618	38.63	0.9887	0.00209	42.76	0.9978
	60	0.0859	55.14	0.9928	0.00239	59.42	0.9948
	90	0.8756	67.52	0.9828	0.00200	72.73	0.9946
Pb^{2+}/Cd^{2+}	30	0.0520	41.15	0.9893	0.00157	46.10	0.9924
	60	0.0577	61.37	0.9934	0.00121	68.19	0.9946
	90	0.0474	85.93	0.9919	0.00064	97.58	0.9958
$Pb^{2+}/Cu^{2+}/Cd^{2+}$	30	0.0442	39.76	0.9914	0.00126	45.43	0.9983
	60	0.1016	40.39	0.9232	0.00339	44.03	0.9717
	90	0.0334	51.96	0.99304	0.00063	61.50	0.9904

Table S3 DB18C6/PA adsorption kinetics fitting parameters for Cu^{2+} in single, binary, and ternary systems

Adsorbates	Concentration (mg/L)	Pseudo-first-order model			Pseudo-second-order model		
		K_1 (min^{-1})	q_e (mg/g)	R^2	K_2 (g/mg·min)	q_e (mg/g)	R^2
Cu^{2+}	30	0.0594	16.43	0.9466	0.00499	18.10	0.9810
	60	0.0308	21.53	0.9872	0.00131	25.91	0.9948
	90	0.0333	25.04	0.9824	0.00125	29.89	0.9874
Cu^{2+}/Pb^{2+}	30	0.0583	10.07	0.9611	0.00773	11.14	0.9900
	60	0.0498	15.08	0.9729	0.00397	16.99	0.9735
	90	0.1563	16.15	0.9089	0.01339	17.34	0.9490
Cu^{2+}/Cd^{2+}	30	0.0760	14.69	0.9693	0.00749	15.99	0.9920
	60	0.0375	18.26	0.9180	0.00250	20.74	0.9508
	90	0.0392	21.24	0.9600	0.00217	24.22	0.9770
$Cu^{2+}/Pb^{2+}/Cd^{2+}$	30	0.0406	10.48	0.9758	0.00424	12.08	0.9964
	60	0.0447	14.37	0.9787	0.00367	16.32	0.9940
	90	0.0442	16.44	0.8574	0.00364	18.32	0.9220

Table S4 DB18C6/PA adsorption kinetics fitting parameters for Cd^{2+} in single, binary, and ternary systems

Adsorbates	Concentration (mg/L)	Pseudo-first-order model			Pseudo-second-order model		
		K_1 (min^{-1})	q_e (mg/g)	R^2	K_2 (g/mg·min)	q_e (mg/g)	R^2
Cd^{2+}	30	0.0258	17.87	0.9710	0.00131	21.71	0.9779
	60	0.0351	18.88	0.9471	0.00205	21.84	0.9692
	90	0.0339	22.34	0.9465	0.00168	25.83	0.9541
Cd^{2+}/Pb^{2+}	30	0.0154	8.505	0.9584	0.00117	11.44	0.9636
	60	0.0254	13.35	0.9765	0.00168	16.33	0.9866
	90	0.0403	14.52	0.9112	0.00346	16.41	0.9534
Cd^{2+}/Cu^{2+}	30	0.0121	13.79	0.9632	0.00367	18.99	0.9566
	60	0.0473	17.04	0.9207	0.00054	18.95	0.9722
	90	0.0300	21.83	0.9135	0.00132	26.12	0.9523
$Cd^{2+}/Pb^{2+}/Cu^{2+}$	30	0.0181	6.597	0.9969	0.00188	8.684	0.9985
	60	0.0176	9.306	0.9910	0.00130	12.25	0.9949
	90	0.1410	12.37	0.9927	0.02287	12.94	0.9938

Langmuir isotherm model (Equation S3) and Freundlich isotherm model (Equation S4) are as follows：

$$q_e = q_m K_L C_e/(1 + bC_e) \qquad \text{（Equation S3）}$$

$$q_e = K_F {C_e}^{1/n} \qquad \text{（Equation S4）}$$

Where q_e is the amount of Pb^{2+}, Cu^{2+}, or Cd^{2+} adsorbed by DB18C6/PA at equilibrium and q_m is saturation capacity of DB18C6/PA forPb^{2+}, Cu^{2+}, or Cd^{2+}， mg/g; C_e is the concentration of Pb^{2+}, Cu^{2+} and Cd^{2+} in the solution at equilibrium, μg/mL; K_L is the adsorption equilibrium constant of Langmuir isothermal model, L/mg; K_F is the adsorption equilibrium constant of Freundlich isothermal model, $mg \cdot g^{-1} \cdot (L \cdot mg^{-1})^{1/n}$; 1/n is the adsorption intensity.

Table S5 DB18C6/PA adsorption isotherm curve fitting parameters for Pb^{2+} in single, binary, and ternary systems

Adsorbates	Temperature (°C)	Langmuir isotherm model			Freundlich isotherm model		
		q_m (mg/g)	K_L	R^2	n	K_F	R^2
Pb^{2+}	25	96.67	0.0780	0.9997	3.17	24.53	0.9571
	35	97.06	0.1103	0.9927	2.70	22.88	0.9124
	45	129.15	0.0821	0.9858	2.64	24.32	0.9305
Pb^{2+}/Cu^{2+}	25	80.74	0.1097	0.9810	3.28	21.01	0.9975
	35	90.85	0.1032	0.9876	3.08	21.78	0.9802
	45	92.30	0.1205	0.9699	3.29	24.80	0.9993
Pb^{2+}/Cd^{2+}	25	87.42	0.1268	0.9748	3.21	23.39	0.9997
	35	90.72	0.1391	0.9501	3.27	25.29	0.9974
	45	91.36	0.1438	0.9818	3.66	29.28	0.9755
$Pb^{2+}/Cu^{2+}/Cd^{2+}$	25	80.60	0.0831	0.9831	3.01	17.60	0.9971
	35	92.65	0.0746	0.9824	2.72	17.40	0.9979
	45	91.72	0.0902	0.9641	2.92	19.96	0.9987

Table S6 DB18C6/PA adsorption isotherm curve fitting parameters for Cu^{2+} in single, binary, and ternary systems

Adsorbates	Temperature (°C)	Langmuir isotherm model			Freundlich isotherm model		
		q_m (mg/g)	K_L	R^2	n	K_F	R^2
Cu^{2+}	25	27.23	0.1399	0.9755	4.54	9.775	0.9756
	35	28.14	0.1528	0.9766	4.61	10.48	0.9519
	45	29.85	0.1497	0.9755	4.36	10.63	0.9773
Cu^{2+}/Pb^{2+}	25	19.92	0.0808	0.8900	2.92	4.048	0.9927
	35	21.68	0.0895	0.9579	2.93	4.575	0.9945
	45	22.14	0.1047	0.9649	3.09	5.156	0.9942
Cu^{2+}/Cd^{2+}	25	21.11	0.2054	0.8903	5.19	8.826	0.9933
	35	22.54	0.2406	0.9280	5.39	9.981	0.9970
	45	23.22	0.3555	0.8700	6.15	11.77	0.9691
$Cu^{2+}/Pb^{2+}/Cd^{2+}$	25	18.41	0.0727	0.8592	2.87	3.542	0.9845
	35	18.32	0.0907	0.8274	3.06	4.076	0.9737
	45	18.83	0.1042	0.8830	3.23	4.626	0.9930

Table S7 DB18C6/PA adsorption isotherm curve fitting parameters for Cd^{2+} in single, binary, and ternary systems

Adsorbates	Temperature (°C)	Langmuir isotherm model			Freundlich isotherm model		
		q_m (mg/g)	K_L	R^2	n	K_F	R^2
Cd^{2+}	25	25.85	0.1281	0.9989	4.75	9.455	0.9651
	35	27.63	0.1267	0.9875	4.58	9.798	0.9895
	45	27.89	0.1564	0.9912	5.12	11.17	0.9843
Cd^{2+}/Pb^{2+}	25	22.03	0.0275	0.9914	1.88	1.521	0.9955
	35	22.75	0.0308	0.9840	1.81	1.556	0.9984
	45	24.14	0.0332	0.9615	1.81	1.746	0.9952
Cd^{2+}/Cu^{2+}	25	23.96	0.1322	0.9638	4.86	9.031	0.9998
	35	25.37	0.1254	0.9301	4.81	9.399	0.9888
	45	26.68	0.1217	0.9302	4.86	9.882	0.9617
$Cd^{2+}/Pb^{2+}/Cu^{2+}$	25	16.01	0.0396	0.9763	2.19	1.693	0.9773
	35	18.35	0.0366	0.9644	1.97	1.594	0.9968
	45	18.92	0.0448	0.9531	2.20	2.14	0.9679

The thermodynamic parameters of the adsorption process are calculated by Equations (S5)-(S8).

$$\ln K = \frac{\Delta S^{\theta}}{R} - \frac{\Delta H^{\theta}}{RT}$$ **(Equation S5)**

$$\Delta G^{\theta} = -RT\ln K$$ **(Equation S6)**

$$\Delta G^{\theta} = \Delta H^{\theta} - \mathrm{T}\Delta S^{\theta}$$ **(Equation S7)**

$$K_{\mathrm{d}} = \frac{q_e}{C_e}$$ **(Equation S8)**

Where R is the ideal gas constant, 8.314 J/(mol • K); T is the absolute temperature, K; ΔG^{θ} is the change of Gibbs free energy, J/K; ΔH^{θ} is the change of enthalpy, J/K; ΔS^{θ} is change of entropy, J/(mol • K); K is the equilibrium constant, (L/mol); q_e is the amount of Pb^{2+}, Cu^{2+} or Cd^{2+} in the DB18C6/PA at equilibrium; C_e is the concentration of Pb^{2+}, Cu^{2+} or Cd^{2+} in the solution at equilibrium, mg/L.

Table S8 Thermodynamic parameters of Pb^{2+}adsorption by DB18C6/PA in single, binary, and ternary systems

Adsorbates	ΔG^{θ} （kJ/mol）			ΔH^{θ} （kJ/mol）	ΔS^{θ} （kJ/mol·K）
	25℃	35 ℃	45 ℃		
Pb^{2+}	-4.576	-4.851	-5.363	7.196	0.0394
Pb^{2+}/Cu^{2+}	-4.206	-4.622	-5.088	8.951	0.0441
Pb^{2+}/Cd^{2+}	-4.796	-5.245	-5.531	6.134	0.0368
$Pb^{2+}/Cu^{2+}/Cd^{2+}$	-3.679	-4.058	-4.526	8.963	0.0424

Table S9 Thermodynamic parameters of Cu^{2+}adsorption by DB18C6/PA in single, binary, and ternary systems

Adsorbates	ΔG^{θ} （kJ/mol）			ΔH^{θ} （kJ/mol）	ΔS^{θ} （kJ/mol·K）
	25℃	35 ℃	45 ℃		
Cu^{2+}	-1.488	-1.847	-2.107	7.723	0.0310
Cu^{2+}/Pb^{2+}	-0.305	-0.763	-1.145	12.205	0.0420
Cu^{2+}/Cd^{2+}	-1.354	-1.989	-2.731	19.191	0.0689
Cu^{2+}/Pb^{2+}/Cd^{2+}	-0.095	-0.238	0.522	6.294	0.0214

Table S10 Thermodynamic parameters of Cd^{2+}adsorption by DB18C6/PA in single, binary, and ternary systems

Adsorbates	ΔG^{θ} （kJ/mol）			ΔH^{θ} （kJ/mol）	ΔS^{θ} （kJ/mol·K）
	25℃	35 ℃	45 ℃		
Cd^{2+}	-1.169	-1.348	-1.631	5.735	0.0231
Cd^{2+}/Pb^{2+}	-0.366	-0.912	-1.356	14.375	0.0495
Cd^{2+}/Cu^{2+}	-0.816	-0.916	-1.029	2.3615	0.0107
$Cd^{2+}/Pb^{2+}/Cu^{2+}$	-0.013	-0.546	-1.051	15.456	0.0519

Distribution coefficient (K_d) and adsorption selectivity factor α_M^A are calculated by Equations (S9)-(S10).

$$K_d = (C_0 - C_e) / C_e \times V / m \qquad \textbf{(Equation S9)}$$

$$\alpha_M^A = \frac{q_A C_M}{q_M C_A} \qquad \textbf{(Equation S10)}$$

Where C_0 is the concentration of heavy metals in the solution before adsorption, mg/mL, C_e is the concentration of heavy metals in solution at adsorption equilibrium, g/ml, V is the volume of sample solution, mL; m is the weight of sample, g; q_A is the adsorption amount of A at adsorption equilibrium, mg/g; q_M is the adsorption amount of M at adsorption equilibrium, mg/g; C_A is the concentration of A in solution at adsorption equilibrium, mg/L; C_M is the concentration of M in solution at adsorption equilibrium, mg/L.

Table S11 Adsorption distribution coefficient and selectivity factor of DB18C6/PA for Pb^{2+}, Cu^{2+} and Cd^{2+} in single, binary and ternary systems

Adsorbate	K_{dA}	K_{dM}	α_M^A
Pb^{2+}	5.4289	/	/
Cu^{2+}	0.9292	/	/
Cd^{2+}	0.7697	/	/
Pb^{2+}/Cu^{2+}	4.0887	0.5822	7.0228
Pb^{2+}/Cd^{2+}	4.9411	0.4111	12.0192
Cu^{2+}/Cd^{2+}	0.9631	0.5848	1.6469
$Pb^{2+}/Cu^{2+}/Cd^{2+}$	3.0490	0.9931	3.0702
$Cu^{2+}/Pb^{2+}/Cd^{2+}$	0.6049	4.0421	0.1496
$Cd^{2+}/Pb^{2+}/Cu^{2+}$	0.3882	3.6539	0.1062

Note: The first metal ion represents the target ion A, the other ions represent the interfering ion M.

Table S12. Comparison of adsorption capacity based on the crown ether-based/functionalized adsorbents

adsorbents	adsorbates	adsorption capacity (mg/g)	ref
Anisotropic crown ether grafted bamboo pulp aerogel (DB18C6/PA)	Pb^{2+}	129.15	this work
Benzo-crown ether-functionalized silica (BCES)	Ca^{2+}	40.6	58
Evaluation of performance of functionalized amberlite (XAD7)	Pd^{2+}	6.5	59
Adsorbent based on crown ether functionalized mesoporous silica (MS-C-D)	Ag^{+}	39.8	60
Graphene oxide functionalized with aza-crown ether (FGO)	Tl^{+}	112.21	61
